# Differences in the clinical characteristics and outcomes of COVID-19 patients in the epicenter and peripheral areas of the pandemic from China: a retrospective, large-sample, comparative analysis

**DOI:** 10.1186/s12879-020-05728-7

**Published:** 2021-02-24

**Authors:** Gang Wang, Feng Ming Luo, Dan Liu, Jia Sheng Liu, Ye Wang, Hong Chen, Pan Wen Tian, Tao Fan, Li Tang, He Yu, Lan Wang, Mei Feng, Zhong Ni, Bo Wang, Zhi Fang Song, Xiao Ling Wu, Hong Jun Wang, Xiang Tong, Miao Xue, Xian Ying Lei, Bo Long, Chao Jia, Jun Xiao, Juan Shang, Nian Xiong, Jian Fei Luo, Zong An Liang, Wei Min Li, on behalf of the Sichuan & Wuhan Collaboration Research Group, and Sichuan Treatment Expert Group for COVID-19, China

**Affiliations:** 1 Department of Respiratory and Critical Care Medicine, Clinical Research Center for Respiratory Disease, West China Hospital, Sichuan University, Chengdu, 610041 Sichuan China; 2 Laboratory of Pulmonary Immunology and Inflammation, Frontiers Science Center for Disease-related Molecular Network, Sichuan University, Chengdu, 610041 Sichuan China; 3 Department of Gastrointestinal Surgery, Renmin Hospital of Wuhan University, Wuhan, 430060 Hubei China; 4 Department of Critical Care Medicine, Public Health Clinical Center of Chengdu, Chengdu, 610061 Sichuan China; 5 Department of Integrated Traditional Chinese and Western Medicine, West China Hospital, Sichuan University, Chengdu, 610041 Sichuan China; 6 Department of Respiratory Medicine, Dazhou Central Hospital, Dazhou, 635000 Sichuan China; 7 Department of Critical Care Medicine, Affiliated Hospital of Southwest Medical University, Luzhou, 646000 Sichuan China; 8 Mianyang 404 Hospital, Mianyang, 621000 Sichuan China; 9 Department of Critical Care Medicine, Mianyang Central Hospital, Mianyang, 621000 Sichuan China; 10 Department of Respiratory and Critical Care Medicine, People’s Hospital of Ganzi Prefecture, Ganzi, 626700 Sichuan China; 11 Department of Critical Care Medicine, Nanchong Central Hospital, Nanchong, 637000 Sichuan China; 12 Department of Neurology, Union Hospital, Tongji Medical College, Huazhong University of Science and Technology, Wuhan, 430022 Hubei China; 13 Wuhan Red Cross Hospital, Wuhan, 430015 Hubei China

**Keywords:** COVID-19, Case fatality, Epicenter, Peripheral area, Pandemic, Comparative analysis

## Abstract

**Background:**

There is limited information on the difference in epidemiology, clinical characteristics and outcomes of the initial outbreak of the coronavirus disease (COVID-19) in Wuhan (the epicenter) and Sichuan (the peripheral area) in the early phase of the COVID-19 pandemic. This study was conducted to investigate the differences in the epidemiological and clinical characteristics of patients with COVID-19 between the epicenter and peripheral areas of pandemic and thereby generate information that would be potentially helpful in formulating clinical practice recommendations to tackle the COVID-19 pandemic.

**Methods:**

The Sichuan & Wuhan Collaboration Research Group for COVID-19 established two retrospective cohorts that separately reflect the epicenter and peripheral area during the early pandemic. The epidemiology, clinical characteristics and outcomes of patients in the two groups were compared. Multivariate regression analyses were used to estimate the adjusted odds ratios (aOR) with regard to the outcomes.

**Results:**

The Wuhan (epicenter) cohort included 710 randomly selected patients, and the peripheral (Sichuan) cohort included 474 consecutive patients. A higher proportion of patients from the periphery had upper airway symptoms, whereas a lower proportion of patients in the epicenter had lower airway symptoms and comorbidities. Patients in the epicenter had a higher risk of death (aOR=7.64), intensive care unit (ICU) admission (aOR=1.66), delayed time from illness onset to hospital and ICU admission (aOR=6.29 and aOR=8.03, respectively), and prolonged duration of viral shedding (aOR=1.64).

**Conclusions:**

The worse outcomes in the epicenter could be explained by the prolonged time from illness onset to hospital and ICU admission. This could potentially have been associated with elevated systemic inflammation secondary to organ dysfunction and prolonged duration of virus shedding independent of age and comorbidities. Thus, early supportive care could achieve better clinical outcomes.

**Supplementary Information:**

The online version contains supplementary material available at 10.1186/s12879-020-05728-7.

## Background

In December 2019, an outbreak of pneumonia of unknown cause was identified in Wuhan, the capital of Hubei province in China. A novel coronavirus, the severe acute respiratory syndrome coronavirus 2 (SARS-CoV-2), which had not been detected previously in humans, was identified subsequently by Chinese scientists as the cause [1]. The disease was named the coronavirus disease 2019 (COVID-19) by the World Health Organization (WHO). The clinical spectrum of COVID-19 appears to be wide, and ranges from self-limited mild upper respiratory tract illness to severe pneumonia causing hospitalization or death. The clinical characteristics of some COVID-19 case series in Wuhan, the epicenter of the pandemic, have been previously reported in detail. The reports indicated that 26 to 33% of patients required intensive care and 4 to 15% died [2–4].

After the outbreak of COVID-19 in Wuhan, the government of the Sichuan province implemented strict measures to combat COVID-19. The Health Commission of Sichuan Province (HCSP) focused on traditional public health outbreak response tactics, including isolation, quarantine, social distancing, and community containment, as recommended by the National Health Commission of China. All medical resources were allocated by the HCSP to ensure efficient use. An expert panel drawn from multidisciplinary teams was established and comprised 125 physicians who were led by Dr. Wei Min Li and Dr. Zong An Liang (the corresponding authors of this study) since January 15, 2020. This expert panel soon released emergency prevention and control guidelines for COVID-19 in the medical institutions of the Sichuan province [5]. Furthermore, we funded two additional important expert panels with psychological counseling [6] and traditional Chinese medicine as complementary and alternative treatment options [7, 8]. Physicians caring for severely or critically ill patients could receive daily internet consultations with members of the expert panel. There were 208 designated hospitals across Sichuan Province that were accessible for SARS-CoV-2-suspected or -confirmed individuals. This arrangement resulted in improved outcomes in Sichuan province, one of the peripheral areas of the pandemic. In other peripheral areas, 2 to 10.1% of patients needing intensive care, and an approximately 1.0% mortality rate were reported in recently published studies [9–11].

The factors underlying the significantly different clinical outcomes between the epicenter and peripheral areas affected by the pandemic remains largely unexplored. Recently, Liang et al. [12] observed the clinical characteristics and outcomes of hospitalized patients with COVID-19who were treated in Hubei (epicenter) or outside Hubei (non-epicenter). However, as theirs is a multicenter study, the possibility of selection bias for the included patients cannot be ruled out. Furthermore, hospitalized patients in Hubei but not in Wuhan, would not be well representative of the first-generation COVID-19 cases. Considering the rapidly increasing number of cases with SARS-CoV-2 infection worldwide, the existing research into the differences between the epicenter and peripheral areas of the pandemic in the clinical characteristics and outcomes of COVID-19 patients was insufficient.

This study could provide information that would be potentially helpful in formulating clinical practice recommendations to tackle the COVID-19 pandemic worldwide.

## Methods

### Study design and subjects

This was a retrospective study based on two cohorts evaluated by the Sichuan and Wuhan Collaboration Research Group for COVID-19, China. The Wuhan cohort, drawn from the epicenter area of the pandemic, was formed using a computer-generated simple random sampling method that was applied to enroll subjects from two designated hospitals, namely the Wuhan Red Cross Hospital and Renmin Hospital of Wuhan University, Wuhan, China. The Sichuan cohort, as the group of patients from the peripheral area of the pandemic, consisted of SARS-CoV-2-confirmed patients who were consecutively recruited from 41 designated hospitals until March 12, 2020. Based on the exposure history, we further divided the Sichuan cohort into two sub-cohorts, with or without Wuhan exposure history. All patients enrolled in this study were diagnosed with COVID-19 according to the interim guidance issued by the National Health Commission of China and the WHO [13]. SARS-CoV-2 infection was confirmed by a positive result on a real-time reverse-transcriptase-polymerase-chain-reaction of nasopharyngeal, pharyngeal, throat-swab or sputum specimens. Some of these patients were included in studies reported by Wei et al. [14], Xiong et al. [15] and Xiong et al. [16]; however, their study purposes are significantly different from those of this study.

### Data collection

The medical records of patients with COVID-19 were reviewed by members of the trained research team. Epidemiological, demographic, clinical, laboratory, radiological, treatment and outcome data were collected by using standardized data collection forms (modified case record form for the clinical characterization of severe acute respiratory infection that was shared by the International Severe Acute Respiratory and Emerging Infection Consortium [ISARIC]) from the electronic medical records. The cutoff date was Mar 12, 2020. We collected details of the exposure history, clinical symptoms and signs, and laboratory findings on admission. Laboratory examinations were performed according to the clinical care needs of the patients. Data on radiological abnormalities were extracted from the selected documentation. Patients were excluded if their medical records were not available. A team of trained researchers abstracted the data and entered the structured spreadsheet. All data were cross-checked.

### Study outcomes

The primary outcomes included death or mechanical ventilation whether or not it involved intensive care unit (ICU) admission. Mechanical ventilation was performed in the ward if ICU admission was not possible due to the overwhelming numbers of COVID-19 patients. The secondary outcomes were the rate of ICU admission, time from illness onset to ICU admission and discharge, length of hospital stay, and duration of viral shedding after COVID-19 onset. Duration of viral shedding was defined to ended when two consecutive negative results with qPCR detection were obtained at time intervals greater than 24 h. The criteria for discharge were absence of fever for at least 3 days, substantial improvement in both lungs on chest computed tomography (CT), clinical remission of respiratory symptoms and comorbidities, and cessation of SARS-Cov-2.

### Statistical analysis

Continuous variables were compared using the Student’s *t* test or the Mann-Whitney *U* test; categorical variables were compared by the chi-square test or Fisher’s exact test as appropriate. Logistic or linear regression was performed to identify clinical variables that were associated with outcomes. The detailed statistical analysis is described in the supplementary data.

## Results

### Epidemiological and clinical characteristics at hospitalization

**Fig. 1:**
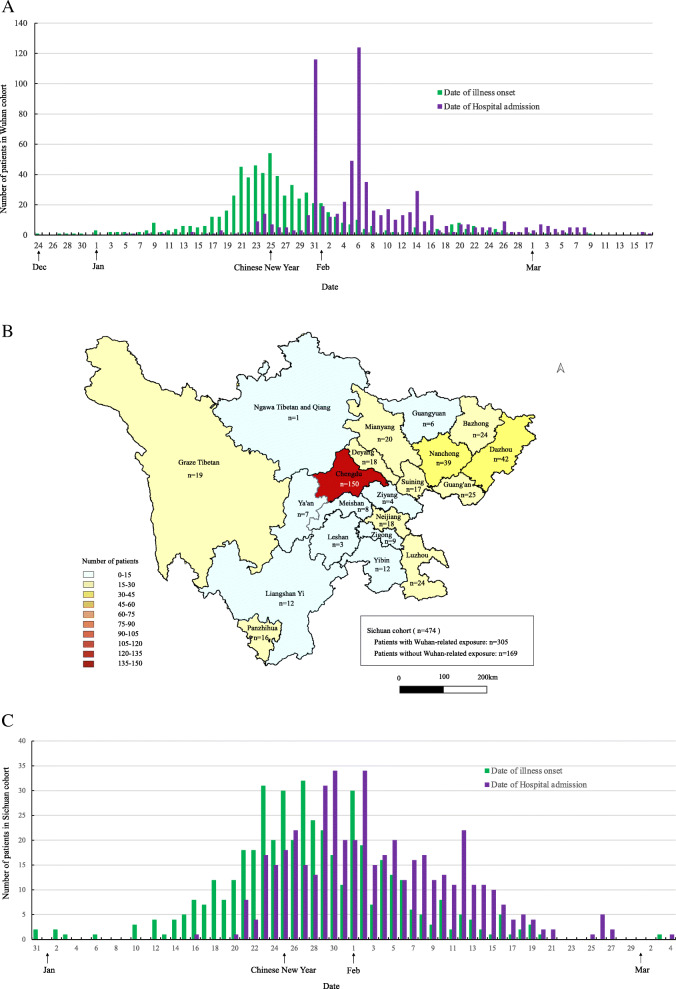
**a** Time of illness onset and hospital admission of patients in the Wuhan cohort; **b** Distribution of patients with COVID-19 in the Sichuan cohort (The data of administrative areas were downloaded from the Database of Global Administrative Areas [GADM] freely available for academic use and we drew this figure using QGIS software version 3.8.3); **c** Time of illness onset and hospital admission of patients in the Sichuan cohort

As of March 12, 2020, a total of 1979 cases from the two hospitals in Wuhan were identified. The Wuhan cohort included 35.9% (*n*=710) of all patients from Wuhan, selected using a computer-generated simple random sampling method, formed the Wuhan cohort. In the Wuhan cohort, illness onset in the first case was noted on December 24, 2019, and the first hospitalization occurred on January 5, 2020 (Fig. 1a).

**Fig. 2:**
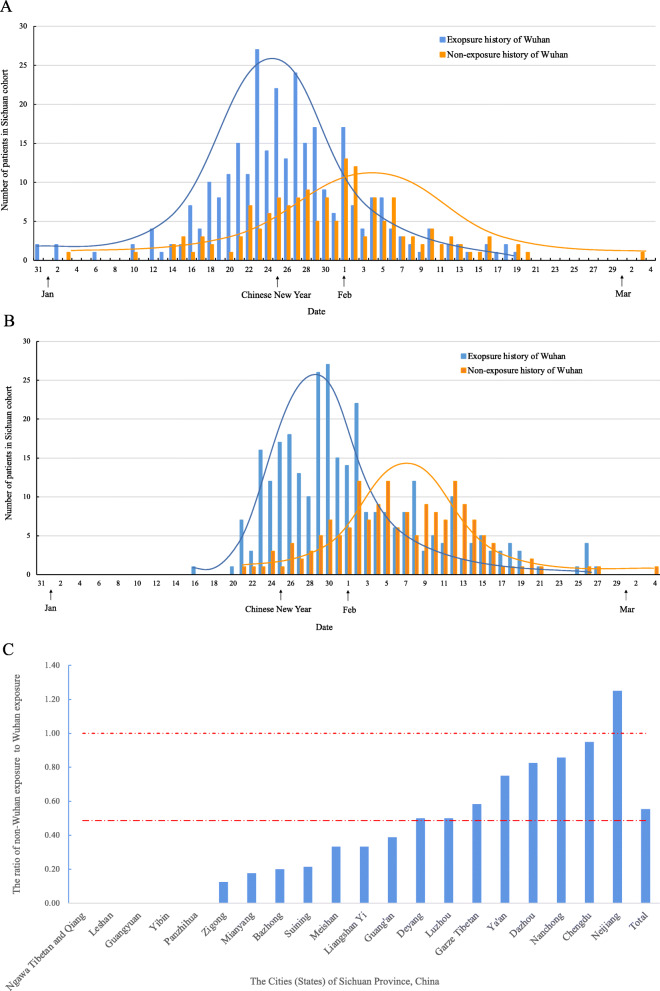
**a** Time of illness onset of patients with or without Wuhan-related exposure in the Sichuan cohort; **b** Time of hospital admission of patient with or without Wuhan-related exposure in the Sichuan cohort; **c** The ratio of the number of patients without Wuhan-related exposure to cases with Wuhan exposure in the Sichuan cohort

There were 538 patients with COVID-19 who were consecutively admitted to 41 designated hospitals in Sichuan Province. The Sichuan cohort comprised 474 patients (Fig. 1b), when 64 patients with inaccessible medical records were excluded. Epidemiological data indicated that the first cases of SARS-CoV-2 infection in the Sichuan cohort occurred in December 31, 2019, and the first case was admitted to the designated hospital on January 16, 2020 (Fig. 1c). The daily Wuhan-related exposure cases with onset of COVID-19 in the Sichuan cohort peaked on January 23, 2020, and those without Wuhan-related exposure peaked on February 1, 2020 (Fig. 2a and b). The median time from illness onset to admission in the Sichuan cohort was significantly shorter than that in the Wuhan cohort (5.0 [2.0, 9.0] vs. 10.0 [7.0, 15.0] days, *P*< 0.001). The Sichuan cohort had a lower proportion of patients with an exposure history than that in the Wuhan cohort (64.3% vs. 99.3%, *P*< 0.001).

The demographic and clinical characteristics of these patients are shown in Table 1 and S1. Patients in the Sichuan cohort were younger (44 [32.0, 54.0] vs. 58 [43.0, 67.0] yrs., *P*< 0.001), there were fewer females (46.4% vs. 54.1%, *P*=0.010), and included a higher number of current smokers (14.5% vs. 5.1%, *P*< 0.001). Two patients (0.4%) in the Sichuan cohort and 13 (1.8%) in the Wuhan cohort were healthcare workers (*P*=0.033). The commonest comorbidity in both cohorts was hypertension (23.6%), followed by diabetes (11.9%). The Wuhan cohort had more cases with comorbidities (51.2% vs. 43.8%, *P*=0.012) as assessed by the Charlson Comorbidity Index [17] (CCI) (2.0 [2.0, 3] vs. 0 [0, 1.0], *P*< 0.001). Fewer patients in the Sichuan cohort had a history of coronary heart disease (*P* =0.004), liver disease (*P*< 0.001), stroke (*P*=0.026), hypertension (*P*< 0.001), and malignancy (*P*=0.012) than those in the Wuhan cohort.

**Table 1:** Demographics and clinical characteristics of patients in the Sichuan and Wuhan cohorts

Variable	Total	Sichuan cohort	Wuhan cohort	χ^2^/Z	*P* value
n	1184	474	710		
Female, n (%)	604 (51.0)	220 (46.4)	384 (54.1)	6.693	0.010
Age, years (Median [IQR])	50.50 (37.00,64.00)	44.00 (32.00,54.00)	58.00 (43.00,67.00)	12.054	< 0.001
Travel in Wuhan/residence in Wuhan/no exposure history, n	141/869/174	128/177/169	13/692/5	527.493	< 0.001
Health care workers, n (%)	15 (1.3)	2 (0.4)	13 (1.8)	4.532	0.033
Current/ ever/ never smoking, n	96/42/892	67/18/376	29/24/516	26.843	< 0.001
Any comorbidity	574 (48.2)	208 (43.8)	366 (51.2)	6.258	0.012
Charlson Comorbidity Index	1.0 (0–2.0)	0 (0–1.0)	2 (2.0–3.0)	−9.190	< 0.001
Disease severity status, n				97.524	< 0.001
Mild/general/severe/critical	194/725/141/127	28/337/81/30	166/388/60/97		
CURB-65 score, n (%)					
0–1/2/3–5	837/68/20	369/12/2	468/56/18	26.430	< 0.001
MuLBSTA score (Median [IQR])	7.00 (5.00,9.00)	5.00 (5.00,9.00)	7.00 (5.00,9.00)	3.96	< 0.001
Laboratory findings, Median (IQR)					
White blood cell count, ×10^9^ /L	5.45 (4.22,7.07)	5.37 (4.18,5.37)	5.58 (4.22,7.28)	1.570	0.088
Neutrophil count, ×10^9^ /L	3.45 (2.50,4.99)	3.45 (2.53,4.73)	3.45 (2.47,5.26)	2.723	< 0.001
Lymphocyte count, ×10^9^ /L	1.18 (0.83,1.63)	1.18 (0.81,1.60)	1.18 (0.84,1.64)	1.074	0.157
Eosinophil count, ×10^9^ /L	0.30 (0.00,1.30)	0.20 (0.01,0.80)	0.50 (0.0,1.7)	5.751	< 0.001
Hemoglobin, g/L	131.00 (119.00,144.00)	137.00 (126.00,151.00)	127.00 (117.00,137.00)	8.049	0.015
Platelet count, ×10^9^ /L	197.00 (148.00,262.50)	175.00 (137.00,230.50)	215.00 (165.00,281.00)	7.909	< 0.001
Activated partial thromboplastin time, s	28.50 (25.90,32.20)	30.90 (27.70,34.90)	27.40 (25.20,29.90)	10.129	< 0.001
Prothrombin time, s	12.20 (11.50,13.00)	12.60 (11.70,13.30)	12.00 (11.33,12.70)	2.052	0.115
D-dimer, mg/L	0.56 (0.29,1.59)	0.50 (0.22,1.17)	0.63 (0.33,1.79)	3.194	0.001
Albumin, g/L	39.70 (35.50,43.30)	43.00 (39.60,45.70)	37.70 (34.00,40.80)	11.556	0.128
Creatinine, μmol/L	63.00 (51.00,76.15)	65.20 (53.00,77.33)	62.00 (50.70,75.00)	2.051	0.040
Creatine kinase, U/L	62.70 (41.00,106.25)	71.00 (50.00,122.00)	56.00 (34.40,99.00)	5.181	< 0.001
Alanine aminotransferase, U/L	23.55 (16.00,39.05)	24.00 (16.00,39.90)	23.00 (15.48,39.00)	0.433	0.665
Aspartate aminotransferase, U/L	25.00 (19.67,35.73)	25.60 (20.00,35.00)	25.00 (19.00,36.00)	0.459	0.647
C-reactive protein, mg/L	19.95 (6.80,53.92)	10.16 (2.67,24.72)	28.45 (8.70,65.20)	6.066	< 0.001
Procalcitonin, ng/mL	0.05 (0.04,0.12)	0.06 (0.04,0.17)	0.05 (0.03,0.11)	2.06	0.039
Hypersensitive troponin I, pg/ml	0.01 (0.01,0.01)	0.01 (0.01,0.01)	0.02 (0.01,0.05)	3.615	< 0.001
Chest CT, n (%)					
Bilateral lungs involvement	741 (93.1)	328 (90.6)	413 (95.2)	6.363	0.012
Consolidation	180 (19.6)	73 (16.0)	107 (23.2)	7.533	0.006
Ground-glass opacity	666 (71.3)	328 (71.6)	338 (71.0)	0.042	0.837
Linear opacity	257 (27.8)	112 (24.5)	145 (31.2)	5.200	0.023
Pleural effusion	49 (5.4)	19 (4.2)	30 (6.6)	2.559	0.110

*CT* Computed tomography

**Fig. 3:**
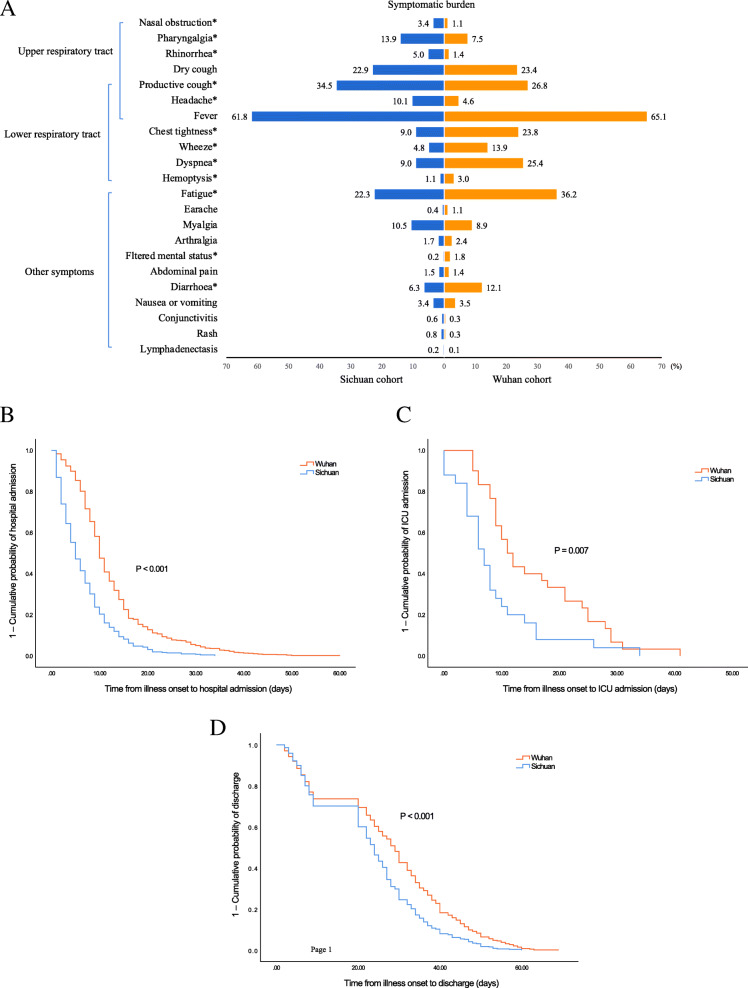
**a** Symptomatic burden of patients with COVID-19 between Sichuan cohort and Wuhan cohorts; Kaplan-Meier survival curve for time from illness onset to hospital admission (**b**), to ICU admission (**c**) and to discharge (**d**) of patients with COVID-19 between Sichuan and Wuhan cohorts

Fever was the commonest symptom and was present in 61.8% of patients in the Sichuan cohort or 65.1% of patients in the Wuhan cohort, but the difference was not significant (*P*=0.246). The Sichuan cohort had a higher incidence of productive cough than the Wuhan cohort (*P*=0.012). However, the Wuhan cohort seemed to have a higher symptomatic burden with regard to the lower respiratory tract, including shortness of breath (25.4% vs. 9.0%, *P*< 0.001), chest distress (23.8% vs. 9.0%, *P*< 0.001), wheeze (13.9% vs. 4.8%, *P*< 0.001), and general symptomatic burden, including fatigue (36.2% vs. 22.3%, *P*< 0.001), hemoptysis (3.0% vs. 1.1%, *P*=0.028), altered consciousness (1.8% vs. 0.2%, *P*=0.011), and diarrhea (12.1% vs. 6.3%, *P*=0.001). In contrast, the Sichuan cohort was more likely to have upper respiratory symptoms, including pharyngalgia (13.9% vs. 7.5%, *P*< 0.001), rhinorrhea (5.0% vs. 1.4%, *P*< 0.001), nasal obstruction (3.4% vs. 1.1%, *P*=0.007), and headache (10.1% vs. 4.6%, *P*< 0.001) (Fig. 3a). Different severity distributions were observed between the two cohorts (*P*< 0.001), as assessed by CURB-65 and MuLBSTA (both *P*< 0.001). More than 75% of patients in both cohorts had mild or general disease, although the Sichuan cohort had a higher proportion of severe cases (17.0% vs. 8.4%) and the Wuhan cohort had more critically ill patients (13.6% vs. 6.3%). Chest CT radiographs in the Wuhan cohort were more likely to show bilateral lung involvement (*P*=0.012) and consolidation (*P*=0.006).

There was no difference in white blood cell count, lymphocyte count, prothrombin time, albumin, alanine aminotransferase, aspartate aminotransferase, procalcitonin and interleukin 6 (IL-6) between the two cohorts. The Sichuan cohort had lower neutrophil count (*P*< 0.001), platelet count (*P*< 0.001), D-dimer levels (*P*=0.001), and C-reactive protein levels (*P*< 0.001) and higher levels of hemoglobin (*P*=0.015), activated partial thromboplastin time (*P*< 0.001), creatinine (*P*=0.040), and creatine kinase (*P*< 0.001) (Table 1).

### Treatments and clinical outcomes

A comparison of treatments and clinical outcomes between the two cohorts is shown in Table 2. Almost all patients received antiviral treatment in Sichuan (94.7%) or Wuhan (93.2%). Fewer patients in the Sichuan cohort received antibiotics (*P*< 0.001), corticosteroids (*P*< 0.001) and supplemental oxygen therapy (*P*< 0.001).

**Table 2:** Treatments and outcomes of the patients in the Sichuan and Wuhan cohorts

Variable	Total	Sichuan cohort	Wuhan cohort	χ^2^/Z	*P* value
n	1184	474	710		
Treatments, n (%)					
Antiviral treatment	1110 (93.8)	448 (94.7)	662 (93.2)	1.067	0.302
Antibiotics	698 (59.0)	204 (43.0)	494 (69.6)	82.734	< 0.001
Antifungal treatment	35 (3.0)	15 (3.2)	20 (2.8)	0.120	0.729
Corticosteroids	272 (23.0)	63 (13.3)	209 (29.4)	41.872	< 0.001
Intravenous immunoglobin	9 (0.8)	3 (0.6)	6 (0.8)	0.170	0.680
Oxygen therapy	791 (66.9)	273 (57.7)	518 (731)	30.175	< 0.001
Prone-position ventilation	30 (2.5)	22 (4.6)	8 (1.1)	14.217	< 0.001
Tracheotomy	8 (0.7)	4 (0.8)	4 (0.6)		0.720*
ECMO	3 (0.3)	1 (0.2)	2 (0.3)		1.000*
Renal replacement	9 (0.8)	5 (1.1)	4 (0.6)		0.497*
Blood transfusion	165 (14.0)	30 (6.3)	135 (19.1)	38.587	< 0.001
Nutrition support	131 (11.1)	52 (11.0)	79 (11.2)	0.014	0.906
TCM treatments	912 (77.0)	418 (88.2)	494 (69.6)	55.620	< 0.001
Physiotherapy	29 (2.4)	24 (5.1)	5 (0.7)	22.605	< 0.001
Outcomes					
Death, n (%)	62 (5.2)	3 (0.6)	59 (8.3)	33.758	< 0.001
ICU admission, n (%)	127 (10.7)	30 (6.3)	97 (13.7)	15.961	< 0.001
Noninvasive mechanical ventilation, n (%)	69 (5.8)	27 (5.7)	42 (5.9)	0.025	0.875
Invasive mechanical ventilation, n (%)	18 (1.5)	8 (1.7)	10 (1.4)	0.148	0.700
Time from illness onset to hospitalization, days	8.00 (4.00,13.00)	5.00 (2.00,9.00)	10.00 (7.00,15.00)	13.626	< 0.001
Hospital stay, days	16.00 (9.00,24.00)	17.00 (12.00,24.00)	14.00 (9.00, 24.00)	−2.726	< 0.001
Time from illness onset to ICU admission, days	9.00 (6.00,17.00)	7.00 (4.00,10.50)	11.50 (8.75,24.25)	3.192^a^	< 0.001
Time from hospitalization to ICU admission, days	3.00 (0.00,9.00)	4.00 (0.00,9.00)	3.00 (0.00,10.50)	0.415	0.678
Time from illness onset to discharge, days	26.00 (18.00,35.00)	23.00 (18.00,31.00)	28.00 (18.00,38.00)	5.693	< 0.001
Time from illness onset to death, days	16.50 (13.00,21.75)	13.00 (11, −)	17.00 (13.00,23.50)	1.240^a^	0.235
Time from hospital admission to death, days	5.00 (3.00,7.00)	10.00 (6.00,-)	4.00 (3.00,7.00)	1.427	0.153
Duration of viral shedding, days	14.00 (9.00, 22.00)	13.00 (8.00,18.00)	17.00 (11.00,27.00)	6.665	< 0.001

* Fisher’s exact test

*ECMO* Extracorporeal membrane oxygenation, *TCM* Traditional Chinese medicine

The case fatality rate in the Sichuan cohort was obviously lower than that in the Wuhan cohort (0.6% vs. 8.3%, *P*< 0.001). However, there was no significant difference in the proportion of patients receiving noninvasive mechanical ventilation or invasive mechanical ventilation between the two cohorts (5.7% vs. 5.9%, *P*=0.872 and 1.7% vs. 1.4%, *P*=0.701). With regard to the secondary outcomes, the proportion of patients who were admitted to the ICU in the Sichuan cohort was significantly lower than that in the Wuhan cohort (6.3% vs. 13.6%, *P*< 0.001). The time from illness onset to ICU admission and time from illness onset to discharge in the Sichuan cohort were shorter than that in the Wuhan cohort (7.0 [4.0, 10.5] vs. 11.5 [8.8, 24.3] days, *P*< 0.001 and 23.0 [18.0, 31.0] vs. 28.0 [18.0, 38.0] days, *P*< 0.001). The length of hospital stay in the Sichuan cohort was much longer than that in the Wuhan cohort (17.0 [12.0, 24.0] vs. 14.0 [9.0, 24.0] days, *P*< 0.001). In contrast, the Wuhan cohort had a significantly prolonged duration of SARS-CoV-2 shedding than that in the Sichuan cohort (19.0 [13.0, 28.0] vs. 14.0 [10.0, 19.0] days, *P*< 0.001).

### Logistic regression analyses

Multivariable logistic regression models were used to explore the differences in clinical outcomes between the Sichuan and Wuhan cohorts (Table 3). The results showed that the Wuhan cohort had higher risk of death (aOR=7.64, 95% CI=[2.31, 25.27], *P*=0.001), ICU admission (aOR=1.66, 95% CI=[1.05, 2.63], *P*=0.031), delayed time from illness onset to hospital (aOR=6.29, 95% CI=[4.70, 8.40], *P*< 0.001) and ICU admission (aOR=8.03, 95% CI=[1.74, 37.06], *P*< 0.001) admissions, prolonged duration of viral shedding after COVID-19 onset (aOR=1.64, 95% CI=[1.15, 2.33], *P*=0.006), a decreased hospital stay (aOR=0.41, 95% CI=[0.32, 0.53], *P*< 0.001) after adjusting for age, sex, smoking status and the CCI. There was no difference in time from illness onset to discharge (aOR=0.99, 95% CI=[0.77, 1.28], *P*=0.968) after adjusting for these confounders. When we additionally adjusted for time from illness onset to hospitalization, the risk of the Wuhan cohort was nearly unchanged; however, the Sichuan cohort had a lower risk for extended time from illness onset to discharge (aOR=0.46, 95% CI=[0.34, 0.63], *P*< 0.001).

**Table 3:** Risk of adverse outcomes in Wuhan cohort in reference to the Sichuan cohort

Outcomes	Unadjusted			Adjusted ^a^		
	OR	95%CI	*P*	OR	95%CI	*P*
Death	14.286	4.444–45.455	< 0.001	7.643	2.311–25.274	0.001
ICU admission	2.347	1.531–3.597	< 0.001	1.659	1.047–2.627	0.031
Non-invasive mechanical ventilation	1.044	0.635–1.718	0.865	0.651	0.376–1.127	0.125
Invasive mechanical ventilation	0.835	0.327–2.132	0.705	0.381	0.138–1.054	0.063
Tracheotomy	0.668	0.166–2.681-	0.569	0.225	0.050–1.015	0.052
Time from illness onset hospitalization (> 5 days)	6.849	5.208–9.009	< 0.001	6.289	4.695–8.403	< 0.001
Hospital stay (> 17 days)	0.481	0.380–0.609	< 0.001	0.411	0.316–0.533	< 0.001
Time from illness onset to ICU admission (> 7 days)	6.364	1.836–22.061	0.027	8.030	1.740–37.057	< 0.001
Time from illness onset to discharge (> 23 days)	1.180	0.935–1.489	0.163	0.995	0.772–1.281	0.968
Time from illness onset to death (> 10 days)	4.706	0.399–55.447	0.218	4.731	0.314–71.265	0.261
Time from hospitalization to ICU admission (> 4 days)	0.857	0.243–3.024	0.811	0.665	0.122–3.620	0.637
Time from hospital admission to death (> 10 days)	0.426	0.035–5.161	0.502	0.155	0.007–3.694	0.249
Duration of viral shedding (> 13 days)	1.881	1.363–2.597	< 0.001	1.640	1.153–2.333	0.006

^a^ Adjusted for sex, age, smoking and Charlson Comorbidity Index

*ICU* Intensive care unit, *CI* Confidence interval, *OR* Odds ratio

In the overall study population of COVID-19 patients from the two cohorts, we constructed multivariable logistic regression models to detect the risk factors at admission for death, ICU admission, mechanical ventilation and duration of viral shedding after COVID-19 onset (Table S2). After adjusting for the cohort sites, sex, age, smoking status and the CCI, we found that white blood cells (> 10× 10^9^/L), neutrophils (> 6.3× 10^9^/L), lymphocytes (> 1.0× 10^9^/L), hemoglobin (< 90 g/L), D-dimer (> 0.5 mg/L), creatine kinase (> 185 IU/L), hyper-sensitive troponin I (> 0.04 ng/mL), alanine aminotransferase (> 50 IU/L), aspartate aminotransferase (> 40 IU/L), procalcitonin (> 0.5 ng/mL) and delayed hospitalization were associated with death, ICU admission and mechanical ventilation. In addition, we found that time from illness onset to hospitalization was associated with prolonged duration of virus shedding (adjusted β=0.11, 95% CI= [0.03, 0.24], *P*=0.009).

We analyzed the relationship between the delay in hospitalization and elevated systemic inflammation and features of organ dysfunction. The time from illness onset to hospitalization was positively correlated with systemic inflammatory cells such as white blood cells (r=0.086, *P*=0.004), neutrophils (r=0.089, *P*=0.003), eosinophils (r=0.116, *P*< 0.001), platelets (r=0.212, *P*< 0.001), and inflammatory biomarkers, such as D-dimer (r=0.101, *P*=0.004), procalcitonin (r=− 0.093, *P*=0.019), features of organ dysfunction, such as hemoglobin (r=− 0.155, *P*< 0.001), BUN (r=0.10, *P*=0.002), creatine (r=− 0.094, *P*=0.003), albumin (r=− 0.263, *P*< 0.001), and APTT (r=− 0.247, *P*< 0.001) (Table 4). After adjusting for age, the correlations of delay in hospitalization with BUN and D-dimer did not achieve statistical significance, which indicated that the delay in hospitalization was independent of age. We further analyzed the relationship between the delay in hospitalization and ICU admission with elevated systemic inflammation after adjusting for age, sex, smoking, and steroid use. In general, the relationship between the delay in hospitalization and elevated systemic inflammation did not change, which implied that these relationships were independent of age, sex, smoking, and steroid use.

**Table 4:** Correlation of time from illness onset to hospitalization with systemic inflammation and features of organ dysfunction

Variables	Model 1		Model 2		Model 3		Model 4	
	r	*P* value	*r*	*P* value	*r*	*P* value	*r*	*P* value
White blood cell count, ×10^9^/L	0.086	0.004	0.081	0.007	0.082	0.007	0.080	0.008
Neutrophil count, × 10^9^/L	0.089	0.003	0.061	0.044	0.064	0.035	0.061	0.046
Lymphocyte count, ×10^9^/L	−0.026	0.391	0.027	0.380	0.025	0.414	0.032	0.293
Eosinophil count, ×10^9^/L	0.116	< 0.001	0.119	< 0.001	0.118	< 0.001	0.127	< 0.001
Hemoglobin, g/L	−0.155	< 0.001	− 0.100	0.001	−0.104	0.001	−0.103	0.001
Platelet count, × 10^9^ /L	0.212	< 0.001	0.225	< 0.001	0.223	< 0.001	0.225	< 0.001
BUN, mmol/L	0.100	0.002	0.038	0.247	0.040	0.215	0.039	0.226
Creatinine, μmol/L	−0.094	0.003	−0.117	< 0.001	−0.141	< 0.001	−0.141	< 0.001
Creatine kinase, U/L	−0.155	< 0.001	−0.145	< 0.001	−0.165	< 0.001	−0.166	< 0.001
Albumin, g/L	−0.263	< 0.001	−0.200	< 0.001	−0.194	< 0.001	−0.192	< 0.001
APTT, s	−0.247	< 0.001	−0.216	< 0.001	−0.212	< 0.001	−0.212	< 0.001
PT, s	−0.018	0.600	−0.019	0.567	−0.021	0.537	−0.021	0.535
D-dimer, mg/L	0.101	0.004	0.043	0.210	0.047	0.175	0.046	0.182
C-reactive protein, mg/L	0.051	0.210	0.021	0.619	0.017	0.674	0.014	0.731
Procalcitonin, ng/mL	−0.093	0.019	−0.123	0.002	−0.130	0.001	−0.135	0.001
IL-6, pg/mL	−0.094	0.407	−0.155	0.171	−0.144	0.211	−0.144	0.215

Model 1: Unadjusted; Model 2: Adjusted for age; Model 3: Adjusted for age, sex, smoking; Model 4: Adjusted for age, sex, smoking, and use steroid use

*APTT* Activated partial thromboplastin time, *PT* Prothrombin time, *BUN* Blood urea nitrogen

### Subgroup analyses between Sichuan sub-cohorts with vs. without Wuhan-related exposure

There was almost no difference in clinical characteristics and outcomes between the two sub-cohorts with and without Wuhan-related exposure in Sichuan. Detailed information is provided in Supplementary Data (Tables S3, S4 and S5).

### Sichuan sub-cohort with Wuhan-related exposure vs. Wuhan cohort

The differences in the clinical characteristics and outcomes between the Sichuan sub-cohort with Wuhan-related exposure and Wuhan cohort were similar to the differences between the Sichuan and Wuhan cohorts. The results are described in detail in the Supplementary Data (Tables S6, S7 and S8).

## Discussion

To the best of our knowledge, there exists a paucity of information obtained from a comparative large-sample study on the differences in epidemiology, clinical characteristics and outcomes of patients with COVID-19 between the epicenter (Wuhan) and the peripheral areas of pandemic. This comparative study provides important insights. First, the outbreak and transmission of COVID-19 within the region of Sichuan as the peripheral epidemic area has been well contained within 2 months through the use of traditional public health outbreak response tactics. Second, the Sichuan cohort is characterized by a higher incidence of upper airway symptoms, whereas the Wuhan cohort was older, had fewer lower airway symptoms and comorbidities, and had elevated pivotal systemic inflammation indicative of organ dysfunction as well as worse clinical outcomes independent of sex, age, smoking and comorbidities. Third, the subgroup analysis indicated that, within the Sichuan cohort, the patients with Wuhan-related exposure had similar clinical features and outcomes to those with non-Wuhan-related exposure. Fourth, the Wuhan-related exposure patients in the Sichuan cohort had better clinical outcomes than those in the Wuhan cohort, although these two groups of patients had a similar Wuhan-related exposure history.

As indicated in recently published studies [12], the COVID-19 patients in Wuhan, at the epicenter area of the epidemic, were older, had more co-existing conditions assessed by the CCI, had extended time from illness onset to hospitalization, and included more severely ill patients. However, the Sichuan cohort, as the peripheral area, had some characteristics features. First, there were fewer healthcare workers in the Sichuan cohort than in the Wuhan cohort, which could be at least partially explained by the insufficient implementation of precautions and the overwhelmed health system during the earlier stage of this outbreak in Wuhan. Second, intriguingly, there was a higher incidence of upper airway symptoms, rather than high incidence of lower airway symptoms in the Wuhan cohort at the epicenter epidemic, which was similar to the findings from exported cases in Singapore [18]. Accordingly, the exported patients from the epicenter were usually diagnosed with a “common cold” at the beginning of the COVID-19 outbreak. The different populations, the airway proliferation location, or the evolution of SARS-CoV-2 possibly could account for these differential symptoms [18–21]. Third, within the consecutively recruited cases in the Sichuan cohort as a well-defined population, the subgroup analyses indicated a higher proportion of males and older patients among the non-Wuhan-related exposure patients, which supported the theory of the propensity for SARS-CoV-2 infection in males and elders [9, 22, 23]. Recent studies from the USA and Italy have reported that a greater proportion of elderly and male COVID-19 patients would experience more critical illness [24, 25].

Until now no antiviral treatment for COVID-19 has proven effective, and supportive care is the mainstay of treatment is. Compared with the Wuhan cohort, the use of antibiotics (i.e. cephalosporin and quinolones) and glucocorticoids in the Sichuan cohort decreased by 26.4 and 16.1%, respectively. These results could possibly be explained as follows. First, as indicated earlier, the expert panel drawn from the multidisciplinary team established by HCSP together developed and adjusted the treatment plan for severely or critically ill patients according to the interim guidance from the National Health Commission of China and the WHO across the 208 designated hospitals in Sichuan by using the 5G network every day. Accordingly, the use of systemic corticosteroids was strictly managed and they were not routinely administered for the treatment of COVID-19 patients. Second, the COVID-19 patients in the Wuhan cohort would actually be more severe or critically ill, which was supported by the increased use of supplemental oxygen in case of acute hypoxia. In addition, prone-position ventilation, physical rehabilitation and a variety of traditional Chinese medicines were used more often in Sichuan under the guidance of the expert panel; however, this aspect needs to be investigated further in randomized controlled trials [7, 8].

In terms of clinical outcomes, several important findings were identified in this study. An epidemic outbreak provided an opportunity to obtain important information, some of which were associated with a limited window of opportunity. This study showed that there was a delay from illness onset to hospitalization in the Wuhan cohort, which might be an important risk factor for the progression of COVID-19. Multivariate regression analysis showed that the time from illness onset to hospitalization was significantly associated with mortality and ICU admission, which suggested some important implications with regard to the pathogenesis of SARS-CoV-2 and may provide insights into a unique window of opportunity for intervention [7]. Liang et al. [12] recently found that Wuhan-related exposure patients have worse clinical outcomes compared with the non-Wuhan-related exposure cases; they attributed the attenuated disease to the onward transmission of SARS-CoV-2. In fact, this is paradoxical to the findings reported from Liang et al.’s study [12] because the relationship between Wuhan-related exposure and prognosis disappeared after adjusting for confounders. Our study firstly found that COVID-19 patients in the Wuhan cohort had worse clinical outcomes including case fatality rate, ICU admission, and duration of virus shedding, independent of sex, age, smoking, comorbidities, and even time from illness onset to hospitalization. The severity of COVID-19 and the shortage of medical resources would partly account for these worse outcomes. For example, during an earlier stage of the outbreak, some patients would not have received sufficient oxygen support because of insufficient oxygen pressure.

The duration of infectious virus replication is an important factor in assessing the risk of transmission and for guiding decisions on the isolation of patients; however, the duration of SARS-CoV-2 RNA detection has not been well explored. Our study found that the Wuhan cohort in the epicenter area had the prolonged virus shedding, which may contribute to the disease severity and clinical course [26, 27]. Furthermore, we found for the first time that the duration of virus shedding was independently associated with age and time from illness onset to hospitalization. Our findings are supported by those of other studies. Liu et al. [28] found that the viral load in severe cases was higher than that of mild cases, which had early viral shedding. Wolfel et al. [29] found that virus shedding in the upper airway, which is the location of mild COVID-19, was very high during the first week of symptoms, whereas shedding of viral RNA from sputum derived from the lower airway, which is the region of general to critical illness in COVID-19, outlasts the disappearance of symptoms. Xu et al. [30] found that elderly patients had prolonged virial shedding, but the correlation of age with the duration of viral shedding disappeared after adjusting for confounders, although this might be partly attributed to the small sample size.

As the pandemic evolves, mutations and natural selection of SARS-CoV-2 inevitably occur, although this virus a lower mutation rate than that of other RNA viruses [31]. The China National Center for Bioinformation aligned 77,801 genome sequences of SARS-CoV-2 that were detected globally and identified a total of 15,018 mutations [32]. Studies have shown that mutations play an important role in the virulence and infectivity of SARS-CoV-2, although no significant association was found between mutations and outcomes pertaining to hospitalization or death [33–35]. Thus, it is unclear whether the different clinical outcomes of patients with COVID-19 between the epicenter and peripheral areas affected by the pandemic are due to mutations in SARS-CoV-2.

This large-sample comparative study provides informative insights into the differences in epidemiology, clinical characteristics and outcomes of patients with COVID-19 between the epicenter (Wuhan) and peripheral (Sichuan) areas of the pandemic. However, there are several limitations that need to be addressed. First, due to the retrospective study design, data generation was clinically driven, and not all laboratory data were available for all patients. Accordingly, the missing data for some patients may have biased the findings. Second, the Sichuan cohort, which represented the peripheral area of the COVID-19 pandemic, was incomplete although consecutive patients accounting for 88.1% of total cases with COVID-19 were recruited from 41 designated hospitals in Sichuan. Third, we did not analyze the genetic diversity of virus strains and the evolutionary history, which might well explain the differences between the epicenter and peripheral areas affected by the pandemic.

## Conclusions

This comparative study found that there were significant differences in the epidemiology, clinical characteristics, and outcomes of patients with COVID-19 between the epicenter and peripheral areas affected by the pandemic. The worse outcomes in the epicenter could be partly explained by the overwhelming of health systems and the delayed time from illness onset to hospitalization. This was associated with elevated systemic inflammation, organ dysfunction and prolonged duration of virus shedding, independent of sex, age, smoking and comorbidities. This has potential implications that are of clinical relevance in interventions for COVID-19. The data suggests that urgent or early supportive care would achieve improved clinical outcomes, leading to a lower death rate although no proven effective therapies currently exist. No differences were found in the epidemiology, clinical characteristics, and outcomes between the first generation and secondary generation of patients in the peripheral area of pandemic. Biological differences accounting for the differences between the Wuhan-related exposure patients in the Sichuan cohort and Wuhan cohort need to be further investigated.

## Supplementary Information

**Additional file 1: Table S1**. Detailed comorbidities of patients in Sichuan and Wuhan cohorts. **Table S2.** Regression analysis of the risk factors for death, ICU admission and mechanical ventilation in all patients from the Sichuan and Wuhan cohorts. **Table S3.** Demographics and clinical characteristics of patients in Sichuan sub-cohorts with vs. without Wuhan-related exposure. **Table S4.** Outcomes of patients in Sichuan sub-cohorts with vs. without Wuhan-related exposure. **Table S5.** Risk of adverse outcomes in Sichuan sub-cohorts with vs. without Wuhan-related exposure. **Table S6.** Demographics and clinical characteristics of patients in Sichuan sub-cohort with Wuhan-related exposure vs. Wuhan cohort. **Table S7.** Outcomes in Sichuan sub-cohort with Wuhan-related exposure vs. Wuhan cohort. **Table S8.** Risk of adverse outcomes in Sichuan sub-cohort with Wuhan-related exposure vs. the Wuhan cohort.

## Data Availability

The datasets used and/or analysed during the current study are available from the corresponding author on reasonable request.

## Abbreviations

aOR: Adjusted odds ratio
CCI: Charlson comorbidity index
CI: Confidence interval
COVID-19: Coronavirus disease 2019
HCSP: The Health Commission of Sichuan Province
ISARIC: International Severe Acute Respiratory and Emerging Infection Consortium
OR: Odds ratio
SARS-CoV-2: Severe acute respiratory syndrome coronavirus 2
WHO: World health organization
