# Supplementary material for: Differences in the clinical characteristics and outcomes of COVID-19 patients in the epicenter and peripheral areas of the pandemic from China: a retrospective, large-sample, comparative analysis
Source: BMC Infect Dis. 2021 Feb 24;21:206. doi: 10.1186/s12879-020-05728-7 (PMC7903397; doi:10.1186/s12879-020-05728-7)
Supplement: Supplementary file 1 — Additional file 1: Table S1. Detailed comorbidities of patients in Sichuan and Wuhan cohorts. Table S2. Regression analysis of the risk factors for death, ICU admission and mechanical ventilation in all patients from the Sichuan and Wuhan cohorts. Table S3. Demographics and clinical characteristics of patients in Sichuan sub-cohorts with vs. without Wuhan-related exposure. Table S4. Outcomes of patients in Sichuan sub-cohorts with vs. without Wuhan-related exposure. Table S5. Risk of adverse outcomes in Sichuan sub-cohorts with vs. without Wuhan-related exposure. Table S6. Demographics and clinical characteristics of patients in Sichuan sub-cohort with Wuhan-related exposure vs. Wuhan cohort. Table S7. Outcomes in Sichuan sub-cohort with Wuhan-related exposure vs. Wuhan cohort. Table S8. Risk of adverse outcomes in Sichuan sub-cohort with Wuhan-related exposure vs. the Wuhan cohort. [file 12879_2020_5728_MOESM1_ESM.doc]

Supplementary Data

**Differences in the clinical characteristics and outcomes of COVID-19 patients in the epicenter and peripheral areas of the pandemic from China: a retrospective, large-sample, comparative analysis**

Gang Wang^1, 2*^, Feng Ming Luo^1, 2*^, Dan Liu^1*^, Jia Sheng Liu^3*^, Ye Wang^1^, Hong Chen^4^, Pan Wen Tian^1^, Tao Fan^5^, Li Tang^1^, He Yu^1^, Lan Wang^1^, Mei Feng^1^, Zhong Ni^1^, Bo Wang^1^, Zhi Fang Song^1^, Xiao Ling Wu^1^, Hong Jun Wang^6^, Xiang Tong^1^, Miao Xue^1^, Xian Ying Lei^7^, Bo Long^8^, Chao Jia^9^, Jun Xiao^10^, Juan Shang^11^, Nian Xiong^12, 13#^, Jian Fei Luo^3#^, Zong An Liang^1#^, and Wei Min Li^1#^, On behalf of the Sichuan & Wuhan Collaboration Research Group, and Sichuan Treatment Expert Group for Covid-19, China.

* These authors contributed equally to this article.

^#^ These senior authors contributed equally to this article.

^1^ Department of Respiratory and Critical Care Medicine, Clinical Research Center for Respiratory Disease, West China Hospital, Sichuan University, Chengdu 610041, Sichuan, China

^2^ Laboratory of Pulmonary Immunology and Inflammation, Frontiers Science Center for Disease-related Molecular Network, Sichuan University, Chengdu 610041, Sichuan, China.

^3^ Department of Gastrointestinal Surgery, Renmin Hospital of Wuhan University, Wuhan 430060, Hubei, China.

^4^ Department of Critical Care Medicine, Public Health Clinical Center of Chengdu, Chengdu 610061, Sichuan, China.

^5^ Department of Integrated Traditional Chinese and Western Medicine, West China Hospital, Sichuan University, Chengdu 610041, Sichuan, China.

^6^ Department of Respiratory Medicine, Dazhou Central Hospital, Dazhou 635000, Sichuan, China.

^7^ Department of Critical Care Medicine, Affiliated Hospital of Southwest Medical University, Luzhou 646000, Sichuan, China.

^8^ Mianyang 404 Hospital, Mianyang 621000, Sichuan, China.

^9^ Department of Critical Care Medicine, Mianyang Central Hospital, Mianyang 621000, Sichuan, China.

^10^ Department of Respiratory and Critical Care Medicine, People's Hospital of Ganzi Prefecture, Ganzi 626700, Sichuan, China.

^11^ Department of Critical Care Medicine, Nanchong Central Hospital, Nanchong 637000, Sichuan, China.

^12^ Wuhan Red Cross Hospital, Wuhan 430015, Hubei, China.

^13^ Department of Neurology, Union Hospital, Tongji Medical College, Huazhong University of Science and Technology, Wuhan 430022, Hubei, China.

**Corresponding Authors**:

Prof. Wei Min Li, MD., Department of Respiratory and Critical Care Medicine, Clinical Research Center for Respiratory Disease, West China Hospital, Sichuan University, Chengdu, Sichuan 610041, China. Email: weimi003@scu.edu.cn

Zong An Liang, MD, Department of Respiratory and Critical Care Medicine, Clinical Research Center for Respiratory Disease, West China Hospital, Sichuan University, Chengdu 610041, Sichuan, China. Email: [liang.zongan@163.com](mailto:liang.zongan@163.com)

Jian Fei Luo, MD, Department of Gastrointestinal Surgery, Renmin Hospital of Wuhan University, Wuhan 430060, Hubei, China. E-mail: [afei099@163.com](mailto:afei099@163.com)

Nian Xiong, MD, Wuhan Red Cross Hospital, Wuhan 430015, Hubei, China. E-mail: [nianxiong@hust.edu.cn](mailto:nianxiong@hust.edu.cn)

**Methods**

**Outbreak response from Sichuan as the peripheral area of pandemic**

All individuals with exposure history, defined as a history of exposure to Wuhan or exposure to infected people within two weeks, were managed by local community to keep them in isolation at least for 14 days. Patients presented with a fever, any respiratory symptoms, or any other symptoms that could not been explained by the known diseases, especially in those with exposure history were sent to the nearest fever clinic to screen SARS-CoV-2 infection with protective equipment in accordance with WHO, the China Centers for Disease Control and Prevention guidelines. Patients who were confirmed with infection of SARS-CoV-2 were admitted to designated hospitals.

**Statistical analysis**

For this study, there were no formal hypotheses being implemented to drive the sample size calculation and we included the maximum number of patients who met the inclusion criteria. We summarized continuous variables as either means and standard deviations or medians with interquartile ranges. We presented categorical variables as count (%) in each category. Continuous variables were compared using the Student's *t* test or Mann-Whitney *U* test depending on parametric or non-parametric data; categorical variables were compared by the chi-square test or Fisher’s exact test. Logistic or linear regression was performed to identify clinical variables that were associated with outcomes. To make our results easier to interpret, we converted continuous variables into categorical variables with cut‐offs based on median values from our cohort. A multivariate regression analysis was used to estimate adjusted odds ratios (aOR) and 95% confidence intervals (CI). The effect of different areas on the time from illness onset to hospitalization, ICU admission or discharge was assessed by means of Kaplan–Meier curves. A two-sided p value < 0.05 was used to determine statistical significance for all tests and 95% CIs were given. The SPSS 21.0 software (IBM Corp, Armonk, NY, USA) was applied for all analyses.

**Results**

**Subgroup analyses between Sichuan sub-cohorts with vs. without Wuhan-related exposure**

64.3% of patients (n=305) had Wuhan exposure history, of whom 128 had recently traveled to Wuhan and 177 lived in Wuhan before travel to Sichuan since December, 2020. The rest of patients (n=169, 35.7%) who had neither contact with Wuhan residents nor visited the city were locally acquired. Accordingly, it formed two sub-cohorts based on an evidence of Wuhan exposure history. Figure 2C shows the ratio of the number of patients without Wuhan-related exposure to patients with Wuhan exposure across cities, which ratio with less than 1.0 indicated a tapered efficiency of person-to-person transmission.

In general, the demographic and clinical characteristics in the sub-cohort without Wuhan-related exposure were slightly different from those with Wuhan-related exposure (Table S3). Patients with Wuhan exposure history were younger than those without Wuhan exposure history (41.0 [30.5, 50.0] vs. 49.0 [36.0, 57.0] yrs, *P*<0.001). Fewer patients with Wuhan exposure history had diabetes (*P*=0.009). Patients with Wuhan exposure history had a higher symptomatic burden including fever (66.2% vs. 54.1%, *P*=0.009), sore throat (17.0% vs. 8.3%, *P*=0.008), which seemed to have upper respiratory symptoms. Patients with Wuhan exposure history showed higher level of creatine kinase (78.0 [55.0, 147.0] vs. 64.2 [44.0, 103.0] μmol/L, *P*=0.013).

There was no significant difference in clinical outcomes including death, mechanical ventilation, ICU admission, time from illness onset to ICU admission, hospital stay, time from illness onset to discharge and duration of viral shedding after Covid-19 onset between these two sub-cohorts (Table S4). The multivariable logistic regression analyses also did not find significant differences in those clinical outcomes even after adjusting for sex, age, smoking status and CCI (Table S5).

**Sichuan sub-cohort with Wuhan-related exposure vs. Wuhan cohort**

Compared with patients with Wuhan-related exposure (n=305) in Sichuan cohort, Wuhan cohort (n=711) was significantly older (41.3 ± 15.3 vs. 55.6 ±16.0 yrs, *P* < 0.001) , to be more female (54.1% vs. 43.0%, *P*=0.001) and more likely to have underlying comorbidities, including hypertension (28.8% vs. 14.4%, *P*<0.001), heart disease (8.9% vs. 4.5%，*P* =0.011), malignancy (3.4% vs. 1.0%，*P*=0.030) and stroke (2.7% vs. 0.7%, *P*=0.038) (Table S6). The Wuhan cohort had a higher proportion of critical (13.6% vs. 6.2%, *P* < 0.001) and mortality rate (8.3% vs. 0.7%, *P* < 0.001). Wuhan cohort had a higher incidence of lower respiratory symptoms including wheeze (14.0% vs. 3.6%, *P*<0.001), dyspnea (25.4% vs. 8.9%, *P*<0.001), chest tightness (23.9% vs. 7.2%, *P*<0.001) and hemoptysis (3.0% vs. 0.3%, *P*=0.008) than those with Wuhan-related exposure in Sichuan cohort. On the contrary, the Wuhan cohort had a lower incidence of upper respiratory symptoms such as pharyngalgia (7.5% vs. 17.0, *P*<0.001), rhinorrhea (1.4% vs. 4.3, *P*=0.005) and nasal obstruction (1.1% vs. 3.3, *P*=0.018). In addition, systemic and local symptoms including fatigue, headache diarrhea, and altered mental status significantly differed between the two groups (Table S6).

There were significant numerous differences in laboratory findings between Wuhan cohort and those with Wuhan-related exposure in Sichuan cohort (Table S6), which included higher neutrophils (3.45 [2.48, 5.26] vs. 3.31 [2.40, 4.46] ×10^9^/L, *P*=0.015), platelet counts (215.0 [166.0, 281.0] vs. 172.0 [137.0, 224.3] ×10^9^/L, *P*<0.001), levels of D-dimer (0.63 [0.33, 1.79] vs. 0.48 [0.21, 1.15] mg/L, *P*=0.003), but lower levels of hemoglobin (127.0 [117.0, 137.0] vs. 140.0 [127, 152] g/L, *P*<0.001), and albumin (37.7 [34.0, 40.8] vs. 43.3 [39.7, 45.9] g/L, *P*<0.001). Besides, patients in Wuhan had higher proportions of abnormalities in chest CT images including bilateral lung involvement of chest CT scan (95.2% vs. 89.7, *P*=0.007) and linear opacity (31.2% vs. 22.9%, *P*=0.013). More patients in Wuhan cohort received antibiotic treatment (69.5% vs. 45.9%, *P* < 0.001), glucocorticoids (29.4% vs. 13.4, *P* < 0.001), oxygen therapy (73.1% vs. 55.6%, *P*< 0.001) and blood transfusion (19.1% vs. 6.9%, *P* < 0.001) compared with patients with Wuhan-related exposure in Sichuan cohort. However, more patients with Wuhan-related exposure in Sichuan cohort were prescribed with TCM treatment (89.5% vs. 69.5%, *P* < 0.001).

Compared with Wuhan cohort, fewer patients with Wuhan-related exposure in Sichuan cohort died (8.3% vs. 0.7%, *P* < 0.001), admitted to ICU (13.6% vs. 6.2%, *P*<0.001) (Table S7). Further, these Wuhan-related exposure patients in Sichuan cohort had shorter time from illness onset to hospital admission (4.0 [2.0, 8.0] vs. 9.0 [4.0, 14.0] days, *P*<0.001), time from illness onset to ICU admission (6.0 [3.5, 8.5] vs. 11.5 [8.8, 24.3] days, *P*=0.001), duration of virus shedding (13.0 [8.0, 18.0] vs. 17.0 [11.0, 27.0] days, *P*=0.001) and extended hospital stay (17.0 [12.0, 23.0] vs. 11.0 [5.0, 21.0] days, *P*<0.001). However, there was no difference in mechanical ventilation and time from illness onset to discharge between the Wuhan cohort and the Wuhan-related exposure patients of Sichuan cohort (Table S7).

The multivariable logistic regression models showed (Table S8), compared with Sichuan cohort with Wuhan-related exposure, Wuhan cohort had increased risk of death (aOR=5.34, 95%CI=[1.24, 22.89], *P*=0.024), delayed time from illness onset to hospital admission (aOR=6.29, 95%CI=[4.51, 8.71], *P*<0.001) and to ICU admission (aOR=28.53, 95%CI-[2.39, 340.80], *P*=0.008), and prolonged duration of viral shedding after onset (aOR=1.64, 95%CI=[1.09, 2.46], *P*=0.018), but shorter hospital stay (aOR=0.42, 95%CI=[0.31, 0.57], *P*<0.001) after adjusted for sex, age, smoking, and CCI (Table S8).

**Table S1. Detailed comorbidities of patients in Sichuan and Wuhan cohorts**

| Variable | Total | Sichuan cohort | Wuhan cohort | χ^2^ | *P* value |
| --- | --- | --- | --- | --- | --- |
| n | 1184 | 474 | 710 |  |  |
| Asthma, n (%) | 9 (0.8) | 5 (1.1) | 4 (0.6) | 0.925 | 0.336 |
| Coronary heart disease, n (%) | 84 (7.1) | 21 (4.4) | 63 (8.8) | 8.384 | 0.004 |
| Chronic obstructive pulmonary disease, n (%) | 24 (2.0) | 9 (1.9) | 15 (2.1) | 0.060 | 0.807 |
| Chronic lung disease, n (%) | 35 (2.9) | 15 (3.2) | 20 (2.8) | 0.130 | 0.718 |
| Chronic kidney disease, n (%) | 24 (2.0) | 12 (2.5) | 12 (1.7) | 1.039 | 0.308 |
| Liver disease, n (%) | 90 (7.6) | 56 (11.7) | 34 (4.7) | 25.018 | <0.001 |
| Nervous system disorder, n (%) | 9 (0.8) | 4 (0.8) | 5 (0.7) | 0.078 | 0.781 |
| Cancer, n (%) | 29 (2.4) | 5 (1.1) | 24 (3.4) | 6.372 | 0.012 |
| Diabetes, n (%) | 142 (11.9) | 48 (10.1) | 94 (13.1) | 2.530 | 0.282 |
| Immune disease, n (%) | 19 (1.6) | 7 (1.5) | 12 (1.7) | 0.076 | 0.783 |
| Dementia, n (%) | 8 (0.7) | 2 (0.4) | 6 (0.8) | 0.747 | 0.387 |
| Malnutrition, n (%) | 3 (0.3) | 2 (0.4) | 1 (0.1) | 0.897 | 0.343 |
| Hematological system diseases, n (%) | 7 (0.6) | 4 (0.8) | 3 (0.4) | 0.871 | 0.351 |
| Stroke, n (%) | 23 (1.9) | 4 (0.8) | 19 (2.7) | 4.962 | 0.026 |
| Hypertension, n (%) | 281 (23.6) | 76 (16.0) | 205 (28.7) | 25.405 | <0.001 |

Table S2. Regression analysis of the risk factors for death, ICU admission and mechanical ventilation in all patients from the Sichuan and Wuhan cohorts ^a^

| Variables | Death | | | ICU admission | | | Non-invasive mechanical ventilation | | | Invasive mechanical ventilation | | |
| --- | --- | --- | --- | --- | --- | --- | --- | --- | --- | --- | --- | --- |
|  | aOR | 95% CI | P | aOR | 95% CI | P | aOR | 95% CI | P | aOR | 95% CI | P |
| White blood cell count, ×10^9^/L |  |  |  |  |  |  |  |  |  |  |  |  |
| <4 | 0.77 | 0.31-1.89 | 0.563 | 0.72 | 0.41-1.27 | 0.261 | 0.50 | 0.21-1.21 | 0.123 | 0.87 | 0.23-3.23 | 0.829 |
| 4~10 | - | - | - | - | - | - | - | - | - | - | - | - |
| >10 | 8.74 | 4.10-18.63 | <0.001 | 3.35 | 1.86-6.02 | <0.001 | 5.00 | 2.51-9.80 | <0.001 | 0.74 | 0.09-6.09 | 0.777 |
| Neutrophil count, ×10^9^/L |  |  |  |  |  |  |  |  |  |  |  |  |
| <1.8 | 0.87 | 0.19-3.91 | 0.851 | 1.50 | 0.73-3.08 | 0.270 | 1.41 | 0.48-4.19 | 0.533 | 1.25 | 0.15-10.21 | 0.839 |
| 1.8~6.3 | - | - | - | - | - | - | - | - | - | - | - | - |
| >6.3 | 7.93 | 4.13-15.24 | <0.001 | 4.79 | 3.02-7.59 | <0.001 | 6.49 | 3.63-11.59 | <0.001 | 1.59 | 0.48-5.33 | 0.451 |
| Lymphocyte count, <1.0×10^9^/L | 0.09 | 0.04-0.21 | <0.001 | 0.36 | 0.24-0.55 | <0.001 | 0.21 | 0.11-0.39 | <0.001 | 0.14 | 0.03-0.66 | 0.013 |
| Haemoglobin, <90 g/L | 7.35 | 2.63-20.53 | <0.001 | 2.64 | 1.07-6.52 | 0.035 | 2.98 | 0.98-9.07 | 0.055 | 3.42 | 0.54-21.74 | 0.193 |
| Platelet count, <100×10^9^/L | 2.35 | 0.83-6.69 | 0.109 | 0.51 | 0.17-1.51 | 0.223 | 0.41 | 0.09-1.79 | 0.234 | 0.61 | 0.07-5.22 | 0.649 |
| D-dimer, ≥0.5mg/L | 5.69 | 1.97-16.47 | 0.001 | 2.72 | 1.59-4.68 | <0.001 | 3.39 | 1.58-7.25 | 0.002 | 3.86 | .79-18.86 | .095 |
| Creatinine, >133 μmol/L | 5.45 | 1.87-15.94 | 0.002 | 2.33 | 0.89-6.06 | 0.084 | 1.19 | 0.31-4.52 | 0.800 | 0.74 | 0.08-6.71 | 0.790 |
| Creatine kinase, >185U/L | 10.23 | 4.41-23.73 | <0.001 | 3.17 | 1.72-5.82 | <0.001 | 4.23 | 2.09-8.55 | <0.001 | 2.33 | 0.63-8.54 | 0.204 |
| Hypersensitive troponin I, >0.04 pg/ml | 13.33 | 5.06-35.12 | <0.001 | 3.89 | 1.60-9.48 | 0.003 | 2.60 | 0.76-8.84 | 0.128 | 10.24 | 0.82-127.94 | 0.071 |
| Alanine aminotransferase, >50 U/L | 2.63 | 1.22-5.69 | 0.014 | 1.70 | 1.01-2.85 | 0.045 | 1.74 | 0.89-3.41 | 0.103 | 1.28 | 0.33-4.94 | 0.719 |
| Aspartate aminotransferase, >40 U/L | 7.11 | 3.60-14.06 | <0.001 | 2.36 | 1.48-3.77 | <0.001 | 2.73 | 1.51-4.96 | 0.001 | 2.21 | 0.67-7.27 | 0.193 |
| CRP, ≥10 mg/L | - | - | - | 2.18 | 1.22-3.88 | 0.008 | 9.97 | 2.32-42.91 | 0.002 | - | - | - |
| Procalcitonin, ≥0.5 ng/mL | 23.87 | 7.62-74.75 | <0.001 | 1.72 | 0.73-4.08 | 0.217 | 1.01 | 0.31-3.25 | 0.991 | 4.26 | 0.88-20.71 | 0.072 |
| Chest CT |  |  |  |  |  |  |  |  |  |  |  |  |
| Bilateral lungs involvement | 0.37 | 0.04-3.17 | 0.363 | 1.41 | 0.42-4.75 | 0.577 | 1.31 | 0.30-5.69 | 0.723 | 0.31 | 0.03-2.91 | 0.307 |
| Consolidation | 0.84 | 0.23-3.10 | 0.794 | 1.09 | 0.63-1.89 | 0.771 | 1.53 | 0.78-2.99 | 0.214 | 1.04 | 0.21-5.02 | 0.965 |
| Ground-glass opacity | 7.33 | 0.94-57.28 | 0.057 | 0.83 | 0.51-1.34 | 0.437 | 1.11 | 0.58-2.15 | 0.749 | 4.11 | 0.50-33.46 | 0.187 |
| Linear opacity | 0.54 | 0.17-1.73 | 0.299 | 0.40 | 0.22-0.72 | 0.002 | 0.47 | 0.22-1.00 | 0.005 | 1.69 | 0.48-5.98 | 0.413 |
| Pleural effusion | 2.57 | 0.63-10.43 | 0.186 | 4.87 | 2.49-9.55 | <0.001 | 6.92 | 3.26-14.70 | <0.001 | 5.18 | 1.19-22.58 | 0.029 |
| Time from illness onset to hospital admission (>5 days) | 2.40 | 1.00-5.78 | 0.049 | 1.42 | 0.60-3.37 | 0.424 | 1.62 | 0.86-3.03 | 0.133 | 1.34 | 0.44-4.09 | 0.609 |
| ^a^ Adjusted for study region, sex, age, smoking, and Charlson Comorbidity Index.  ICU, intensive care unit; CI, confidence interval; OR, odds ratio; aOR, adjusted odds ratio | | | | | | | | | | | | |

**Table S3. Demographics and clinical characteristics of** **patients in Sichuan sub-cohorts with vs. without Wuhan-related exposure.**

| Variable | Sichuan sub-cohort with Wuhan-related exposure | Sichuan sub-cohort without Wuhan-related exposure | χ^2^/Z | *P* value |
| --- | --- | --- | --- | --- |
| n | 305 | 169 |  |  |
| Female, n (%) | 174 (57.0) | 80 (47.3) | 4.124 | 0.042 |
| Age, years | 41.00(30.50,50.00) | 49.00(36.00,57.00) | -4.163 | <0.001 |
| Current / Ever/ Never smoking, n | 48/12/235 | 19/6/141 | 2.121 | 0.346 |
| Health care workers, n (%) | 2 (0.7) | 0 (0) | 1.126 | 0.289 |
| Any comorbidity, n (%) | 146 (47.9) | 70 (41.4) | 1.823 | 0.177 |
| Symptoms |  |  |  |  |
| Nasal obstruction, n (%) | 10 (3.3) | 6 (3.6) | 0.033 | 0.857 |
| Pharyngalgia, n (%) | 52 (17.0) | 14 (8.3) | 6.971 | 0.008 |
| Rhinorrhea, n (%) | 13 (4.3) | 11 (6.5) | 1.142 | 0.285 |
| No/ Dry/ Productive Cough, n | 95/94/116 | 69/48/52 | 4.776 | 0.092 |
| Wheeze, n (%) | 11 (3.6) | 12 (7.1) | 2.876 | 0.090 |
| Chest tightness, n (%) | 22 (7.2) | 21 (12.4) | 3.582 | 0.058 |
| Dyspnea, n (%) | 27 (8.9) | 16 (9.5) | 0.050 | 0.823 |
| Hemoptysis, n (%) | 1 (0.3) | 4 (2.4) |  | 0.057* |
| Fever ( >37.3℃), n (%) | 202 (66.2) | 92 (54.1) | 6.790 | 0.009 |
| Fatigue, n (%) | 73 (23.9) | 33 (19.5) | 1.217 | 0.270 |
| Headache, n (%) | 29 (9.5) | 19 (11.2) | 0.359 | 0.549 |
| Earache, n (%) | 2 (0.7) | 0 (0) |  | 0.542* |
| Myalgia, n (%) | 31 (10.2) | 19 (11.2) | 0.134 | 0.714 |
| Arthralgia, n (%) | 4 (1.3) | 4 (2.4) |  | 0.464* |
| Fltered mental status, n (%) | 0 (0) | 1 (0.6) |  | 0.357* |
| Abdominal pain, n (%) | 5 (1.6) | 2 (1.2) |  | 1.000* |
| Diarrhoea, n (%) | 16 (5.3) | 14 (8.3) | 1.644 | 0.200 |
| Nausea or vomiting, n (%) | 10 (3.3) | 6 (3.6) | 0.025 | 0.875 |
| Conjunctivitis, n (%) | 2 (0.7) | 1 (0.6) |  | 1.000* |
| Rash, n (%) | 1 (0.3) | 3 (1.8) |  | 0.132* |
| Lymphadenectasis, n (%) | 0 (0) | 1 (0.6) |  | 0.357* |
| Disease severity status, n |  |  | 1.900 | 0.593 |
| Mild/ General/ Severe/ Critical | 16/223/47/19 | 11/114/33/11 |  |  |
| Curb-65 score | 0.15±0.41 | 0.37±0.65 | -3.618 | <0.001 |
| MuLBSTA score | 7.00(5.00,9.00) | 5.00(5.00,9.00) | -1.340 | 0.180 |
| Laboratory findings |  |  |  |  |
| White blood cell count, × 10⁹ / L | 5.11(4.00,6.56) | 5.63(4.43,7.44) | -1.771 | 0.077 |
| Neutrophil count, × 10⁹ /L | 67.3(56.6,74.2) | 67.6(58.5,77.75) | -1.837 | 0.067 |
| Lymphocyte count, × 10⁹ /L | 22.7(16.62,30.6) | 23.85(15.475,31.125) | 0.480 | 0.631 |
| Eosnophil count，× 10⁹ /L | 0.20(0.00,0.70) | 0.25(0.04,0.825) | -0.395 | 0.693 |
| Hemoglobin, g/L | 140.00(127.00,152.00) | 134.00(124.00,148.00) | 1.796 | 0.073 |
| Platelet count, × 10⁹/L | 172.00(137.00,224.25) | 180.00(134.00,244.00) | -1.846 | 0.066 |
| Activated partial thromboplastin time, s | 31.3(28.1,34.90) | 30.25(26.7,34,85) | 0.697 | 0.486 |
| Prothrombin time, s | 12.6(11.75,13.40) | 12.3(11.70,13.20) | 0.842 | 0.40 |
| D-dimer, mg/L | 0.50(0.24,1.18) | 0.485(0.2075,1.0175) | 0.241 | 0.809 |
| Albumin, g/L | 43.3(39.65,45.95) | 42.7(39.33,45.20) | 0.141 | 0.888 |
| Creatinine, μmol/L | 67.7(53.1,78.00) | 63.9(52.75,75.4) | -0.395 | 0.693 |
| Creatine kinase, U/L | 78.00(55.00,147.00) | 64.2(44.00,103.00) | 2.507 | 0.013 |
| Alanine aminotransferase, U/L | 23.00(16.00,37.00) | 25.2(16.25,46.00) | -1.271 | 0.205 |
| Aspartate aminotransferase, U/L | 26.00(20.00,34.225) | 25.00(19.3,38.00) | 0.093 | 0.926 |
| C-reactive protein, mg/L | 3.6(5.188,27.20) | 7.81(2.17,22.34) | 0.195 | 0.846 |
| Procalcitonin, ng/mL | 0.0705(0.04,0.2155) | 0.05(0.03,0.078) | 1.408 | 0.161 |
| IL-6, pg/mL | 5.51(1.97,39.79) | 15.49(4.898,25.395) | -0.213 | 0.833 |
| Chest CT |  |  |  |  |
| Bilateral lungs involved, n (%) | 208 (89.7) | 120 (92.3) | 0.689 | 0.407 |
| Consolidation, n (%) | 51 (17.4) | 22 (13.5) | 1.190 | 0.275 |
| Ground-glass opacity, n (%) | 202 (68.7) | 126 (77.3) | 3.822 | 0.051 |
| Linear opacity, n (%) | 67 (22.9) | 45 (27.4) | 1.188 | 0.276 |
| Pleural effusion, n (%) | 10 (3.4) | 9 (5.5) | 1.087 | 0.297 |
| * Fisher's exact test.  CT, computed tomography. | | | | |

**Table S4. Outcomes of patients in Sichuan sub-cohorts with vs. without Wuhan-related exposure.**

| Variable | Sichuan sub-cohort with Wuhan-related exposure | Sichuan sub-cohort without Wuhan-related exposure | χ^2^/Z | P |
| --- | --- | --- | --- | --- |
| n | 305 | 169 |  |  |
| Death, n (%) | 2 (0.7) | 1 (0.6) | 0.009 | 0.925 |
| ICU admission, n (%) | 19 (6.2) | 11 (6.4) | 0.008 | 0.930 |
| Non-invasive mechanical ventilation, n (%) | 18 (5.9) | 9 (5.3) | 0.083 | 0.773 |
| Invasive mechanical ventilation, n (%) | 5 (1.6) | 3 (1.8) | 0.009 | 0.925 |
| Tracheotomy, n (%) | 2 (0.7) | 2 (1.2) | 0.347 | 0.556 |
| Time from illness onset to hospital admission | 4.00(2.00,8.00) | 5.00(1.00,10.00) | -0.510 | 0.610 |
| Hospital stay, days | 17.00(13.00,24.00) | 17.00(13.00,24.00) | -0.677 | 0.499 |
| Time from illness onset to ICU admission, days | 6.00(3.50,8.50) | 8.00(6.00,14.00) | -1.586 | 0.126 |
| Time from illness onset to discharge, days | 23.00(18.00,31.00) | 25.00(19.00,31.00) | -0.835 | 0.404 |
| Duration of viral shedding, days | 14.00(10.00,19.00) | 14.00(11.00,19.50) | -0.0187 | 0.852 |

**Table S5. Risk of adverse outcomes in Sichuan sub-cohorts with vs. without Wuhan-related exposure*.**

| Outcomes | β | SE | aOR | 95%CI | *P* |
| --- | --- | --- | --- | --- | --- |
| ICU admission | -2.111 | 1.745 | 0.121 | 0.004-3.705 | 0.226 |
| Non-invasive mechanical ventilation | -0.380 | 0.453 | 0.684 | 0.282-1.661 | 0.401 |
| Invasive mechanical ventilation | -1.497 | 0.985 | 0.224 | 0.033-1.542 | 0.128 |
| Tracheotomy | -1.159 | 1.431 | 0.314 | 0.019-5.180 | 0.418 |
| Hospital stay (>17 days) | -0.092 | 0.414 | 0.912 | 0.618-1.347 | 0.643 |
| Time from illness onset to ICU admission (>7 days) | -0.287 | 1.016 | 0.750 | 0.102-5.493 | 0.777 |
| Time from hospital admission to ICU admission (>4 days) | -78.389 | 23430.584 | 0.000 | 0.000-- | 0.997 |
| Time from illness onset to discharge (>23 days) | 0.031 | 0.217 | 1.031 | 0.673-1.579 | 0.888 |
| Duration of viral shedding | 0.093 | 0.235 | 0.157 | 0.692-1.740 | 0.692 |
| * Patients with Wuhan-related exposure as reference.  Adjusted for sex, age, smoking, Charlson Comorbidity Index, time from illness onset hospitalization. | | | | | |

**Table S6. Demographics and clinical characteristics of patients in Sichuan sub-cohort with Wuhan-related exposure vs. Wuhan cohort.**

| Variable | Total | Patients with Wuhan-related exposure in the Sichuan cohort (n=305) | Wuhan cohort (n=710) | χ^2^/Z | *P* value |
| --- | --- | --- | --- | --- | --- |
| n | 1015 | 305 | 710 |  |  |
| Female, n (%) | 515 (50.7) | 131 (43.0) | 384 (54.1) | 10.581 | 0.001 |
| Age, years | 50.77±17.17 | 41.29±15.26 | 55.61±15.99 | -12.069 | <0.001 |
| Travel in Wuhan/ Residents in Wuhan/ No exposure history, n | 141/869/6 | 128/177/0 | 13/692/6 | 273.140 | <0.001 |
| Health care workers, n (%) | 15 (1.5) | 2 (0.7) | 13 (1.8) | 2.018 | 0.155 |
| Current/ Ever/ Never smoking, n | 77/36/751 | 48/12/235 | 29/24/516 | 29.948 | <0.001 |
| Any comorbidity, n (%) | 493 (48.5) | 159 (52.1) | 334 (47.0) | 2.271 | 0.132 |
| Charlson Comorbidity Index (CCI) | 0 (0, 1) | 0 (0, 1) | 0 (0, 1) | -0.282 | 0.778 |
| Symptoms |  |  |  |  |  |
| Nasal obstruction, n (%) | 18 (1.8) | 10 (3.3) | 8 (1.1) | 5.639 | 0.018 |
| Pharyngalgia, n (%) | 105 (10.4) | 52 (17.0) | 53 (7.5) | 20.984 | <0.001 |
| Rhinorrhea, n (%) | 23 (2.3) | 13 (4.3) | 10 (1.4) | 7.802 | 0.005 |
| No/ Dry/ Productive Cough, n | 362/339/312 | 95/94/116 | 267/245/196 | 10.896 | 0.004 |
| Wheeze, n (%) | 110 (10.9) | 11 (3.6) | 99 (14.0) | 23.712 | <0.001 |
| Chest tightness, n (%) | 191 (18.9) | 22 (7.2) | 169 (23.9) | 38.657 | <0.001 |
| Dyspnea, n (%) | 207 (20.4) | 27 (8.9) | 180 (25.4) | 36.004 | <0.001 |
| Hemoptysis, n (%) | 22 (2.2) | 1 (0.3) | 21 (3.0) | 6.983 | 0.008 |
| Fever (>37.3℃), n (%) | 664 (65.5) | 202 (66.2) | 462 (65.3) | 0.090 | 0.764 |
| Fatigue, n (%) | 330 (32.6) | 73 (23.9) | 257 (36.3) | 14.839 | <0.001 |
| Headache, n (%) | 62 (6.1) | 29 (9.5) | 33 (4.7) | 8.646 | 0.003 |
| Earache, n (%) | 10 (1.0) | 2 (0.7) | 8 (1.1) |  | 0.732* |
| Myalgia, n (%) | 94 (9.3) | 31 (10.2) | 63 (8.9) | 0.406 | 0.524 |
| Arthralgia, n (%) | 21 (2.1) | 4 (1.3) | 17 (2.4) | 1.247 | 0.264 |
| Fltered mental status, n (%) | 13 (1.3) | 0 (0) | 13 (1.8) |  | 0.013* |
| Abdominal pain, n (%) | 15 (1.5) | 5 (1.6) | 10 (1.4) |  | 0.781* |
| Diarrhea, n (%) | 102 (10.1) | 16 (5.3) | 86 (12.1) | 11.029 | 0.001 |
| Nausea or vomiting, n (%) | 35 (3.5) | 10 (3.3) | 25 (3.5) | 0.041 | 0.840 |
| Conjunctivitis, n (%) | 4 (0.4) | 2 (0.7) | 2 (0.3) |  | 0.588* |
| Rash, n (%) | 3 (0.3) | 1 (0.3) | 2 (0.3) |  | 1.000* |
| Lymphadenectasis, n (%) | 1 (0.1) | 0 (0) | 1 (0.1) |  | 1.000* |
| Disease severity status, n (%) |  |  |  | 71.369 | <0.001 |
| Mild/ General/ Severe/ Critical | 182/611/107/116 | 16/223/47/19 | 166/388/60/97 |  |  |
| Curb-65 score, n |  |  |  | 29.971 | <0.001 |
| 0-1/ 2/ 3-5 | 713/58/19 | 245/2/1 | 468/56/18 |  |  |
| MuLBSTA score | 7 (5, 9) | 7 (5, 9) | 7 (5, 9) | 0.714 | 0.398 |
| Laboratory findings |  |  |  |  |  |
| White blood cell count, × 10⁹ / L | 5.43(4.16, 7.01) | 5.11(4.00, 6.56) | 5.58(4.23, 7.28) | -2.987 | 0.003 |
| Neutrophil count, × 10⁹ /L | 3.40(2.47, 4.95) | 3.31(2.40, 4.46) | 3.45(2.48, 5.26) | -2.421 | 0.015 |
| Lymphocyte count, × 10⁹ /L | 1.17(0.83, 1.61) | 1.17(0.79, 1.58) | 1.18(0.84, 1.63) | -0.691 | 0.490 |
| Eosinophil count，× 10⁹ /L | 0.02(0.00, 0.07) | 0.01(0.00, 0.04) | 0.03(0.00, 0.10) | -5.136 | <0.001 |
| Hemoglobin, g/L | 130.00(119.00, 143.00) | 140.00(127.00, 152.00) | 127.00(117.00, 137.00) | -9.867 | <0.001 |
| Platelet count, × 10⁹/L | 199.00(150.00, 263.50) | 172.00(137.00, 224.25) | 215.00(166.00, 281.00) | -7.651 | <0.001 |
| Activated partial thromboplastin time, s | 28.30(25.80, 31.70) | 31.30(28.10, 34.90) | 27.30(25.17, 29.83) | 4.899 | <0.001 |
| Prothrombin time, s | 12.10(11.50, 13.00) | 12.60(11.75, 13.40) | 12.00(11.30, 12.70) | 3.328 | <0.001 |
| D-dimer, mg/L | 0.57(0.29, 1.66) | 0.48(0.21, 1.15) | 0.63(0.33, 1.79) | -2.986 | 0.003 |
| Albumin, g/L | 39.20(35.20, 42.90) | 43.30(39.65, 45.95) | 37.70(34.00, 40.80) | 5.619 | <0.001 |
| Creatinine, μmol/L | 63.00(51.00, 76.20) | 67.70(53.10, 78.00) | 62.00(50.35, 75.00) | -2.201 | 0.028 |
| Creatine kinase, U/L | 62.00(39.00, 107.00) | 78.00(55.00, 147.00) | 56.00(34.40, 99.00) | -5.672 | <0.001 |
| Alanine aminotransferase, U/L | 23.00(15.73, 38.00) | 23.00(16.00, 37.00) | 23.00(15.35, 39.00) | 0.638 | 0.810 |
| Aspartate aminotransferase, U/L | 25.20(19.70, 35.23) | 26.00(20.00, 34.23) | 25.00(19.00, 36.00) | 0.752 | 0.623 |
| C-reactive protein, mg/L | 11.72(5.00, 43.24) | 10.00(5.13, 24.20) | 12.00(5.00, 49.30) | -1.552 | 0.121 |
| Procalcitonin, ng/mL | 0.05(0.03, 0.12) | 0.07(0.04, 0.20) | 0.05(0.03, 0.11) | -4.470 | <0.001 |
| IL-6, pg/mL | 6.05(3.98, 13.26) | 5.51(1.97, 39.79) | 6.23(4.29, 12.72) | 1.074 | 0.199 |
| Chest CT |  |  |  |  |  |
| Bilateral lungs involved, n (%) | 621 (93.2) | 208 (89.7) | 413 (95.2) | 7.275 | 0.007 |
| Consolidation, n (%) | 158 (20.9) | 51 (17.4) | 107 (23.2) | 3.587 | 0.058 |
| Ground-glass opacity, n (%) | 540 (70.1) | 202 (68.7) | 338 (71.0) | 0.459 | 0.798 |
| Linear opacity, n (%) | 212 (28.0) | 67 (22.9) | 145 (31.2) | 6.170 | 0.013 |
| Pleural effusion, n (%) | 40 (5.4) | 10 (3.4) | 30 (6.6) | 3.400 | 0.065 |
| * Fisher's exact test.  CT, computed tomography | | | | | |

**Table S7. Outcomes in Sichuan sub-cohort with Wuhan-related exposure vs. Wuhan cohort.**

| Variable | Total | Sichuan sub-cohort with Wuhan-related exposure | Wuhan cohort | Statistics | P value |
| --- | --- | --- | --- | --- | --- |
| n | 1015 | 305 | 710 |  |  |
| Treatments |  |  |  |  |  |
| Antibiotics, n | 634 (62.4) | 140 (45.9) | 494 (69.5) | 50.574 | <0.001 |
| Antiviral treatment, n (%) | 953 (93.9) | 291 (95.7) | 662 (93.1) | 2.540 | 0.111 |
| Antifungal treatment, n (%) | 30 (3.0) | 10 (3.3) | 20 (2.8) | 0.162 | 0.688 |
| Corticosteroids, n (%) | 250 (24.6) | 41 (13.4) | 209 (29.4) | 29.279 | <0.001 |
| Intravenous immunoglobin, n (%) | 9 (0.9) | 3 (1.0) | 6 (0.8) |  | 1.000* |
| Oxygen therapy, n (%) | 687 (67.8) | 169 (55.6) | 518 (73.1) | 29.749 | <0.001 |
| Prone position ventilation, n (%) | 20 (2.0) | 12 (3.9) | 8 (1.1) | 8.729 | 0.003 |
| Tracheotomy, n (%) | 6 (0.6) | 2 (0.7) | 4 (0.6) |  | 1.000* |
| ECMO, n (%) | 3 (0.3) | 1 (0.3) | 2 (0.3) |  | 1.000* |
| Renal replacement, n (%) | 6 (0.6) | 2 (0.7) | 4 (0.6) |  | 1.000* |
| Blood transfusion, n (%) | 156 (15.4) | 21 (6.9) | 135 (19.1) | 24.361 | <0.001 |
| Nutrition support, n (%) | 109 (10.8) | 30 (9.8) | 79 (11.2) | 0.397 | 0.529 |
| TCM treatments, n (%) | 767 (75.5) | 273 (89.5) | 494 (69.5) | 46.277 | <0.001 |
| Physiotherapy, n (%) | 19 (1.9) | 14 (4.6) | 5 (0.7) | 17.572 | <0.001 |
| Outcomes |  |  |  |  |  |
| Death, n (%) | 61 (6.0) | 2 (0.7) | 59 (8.3) | 23.144 | <0.001 |
| ICU admission, n (%) | 116 (11.4) | 19 (6.2) | 97 (13.6) | 11.598 | 0.001 |
| Non-invasive mechanical ventilation, n (%) | 60 (5.9) | 18 (5.9) | 42 (5.9) | 0.000 | 0.997 |
| Invasive mechanical ventilation, n (%) | 15 (1.5) | 5 (1.6) | 10 (1.4) |  | 0.780* |
| Time from illness onset to hospital admission, days | 7.00(2.00, 12.00) | 4.00(2.00, 8.00) | 9.00(4.00, 14.00) | -7.733 | <0.001 |
| Hospital stay, days | 14.00(8.00, 22.00) | 17.00(12.00, 23.00) | 11.00(5.00, 21.00) | -7.408 | <0.001 |
| Time from illness onset to ICU admission, days | 9.50(6.00, 17.75) | 6.00(3.50, 8.50) | 11.50(8.75, 24.25) | -3.458 | 0.001 |
| Time from illness onset to discharge, days | 23.00(15.00, 34.00) | 22.00(17.00, 31.00) | 24.00(13.00, 35.00) | -0.352 | 0.725 |
| Time from illness onset to death, days | 16.00(13.00, 21.00) | 14.50(9.75, 12.50) | 17.00(13.00, 21.50) | -0.671 | 0.502 |
| Duration of viral shedding, days | 15.00(9.00, 23.00) | 13.00(8.00, 18.00) | 17.00(11.00, 27.00) | -4.991 | 0.001 |
| * Fisher's exact test.  ECMO, extracorporeal membrane oxygenation; TCM, traditional Chinese medicine. | | | | | |

**Table S8. Risk of adverse outcomes in Sichuan sub-cohort with Wuhan-related exposure vs. the Wuhan cohort*.**

| Outcomes | β | SE | aOR | 95%CI | *P* |
| --- | --- | --- | --- | --- | --- |
| Death | 2.069 | 0.790 | 7.917 | 1.683-37.238 | 0.009 |
| Non-invasive mechanical ventilation | -0.378 | 0.346 | 0.685 | 0.347-1.351 | 0.275 |
| Invasive mechanical ventilation | -0.864 | 0.689 | 0.421 | 0.109-1.625 | 0.210 |
| Tracheotomy | -1.383 | 1.077 | 0.251 | 0.030-2.069 | 0.199 |
| ICU admission | 0.725 | 0.292 | 2.065 | 1.166-3.657 | 0.013 |
| Hospital stay (>17 days) | -0.923 | 0.161 | 0.397 | 0.290-0.544 | <0.001 |
| Time from illness onset to ICU admission (>7 days) | 3.552 | 1.437 | 34.897 | 2.089-582.859 | 0.013 |
| Time from hospital admission to ICU admission (>4 days) | 1.131 | 1.199 | 3.099 | 0.296-32.466 | 0.345 |
| Time from illness onset to discharge (>23 days) | -0.863 | 0.186 | 0.422 | 0.293-0.607 | <0.001 |
| Time from illness onset to death (>10 days) | -26.969 | 270.522 | 0.000 | 0.000-- | 0.999 |
| Duration of viral shedding (> 13 days) | 0.448 | 0.220 | 1.564 | 1.016-2.408 | 0.042 |
| * Patients in Sichuan sub-cohort with Wuhan-related exposure as reference.  Adjusted for sex, age, smoking, Charlson Comorbidity Index, and time from illness onset to hospital admission. | | | | | |
